# Dynamics of changes in broiler spatial distribution induced by a robot with autonomous navigation along the growing cycle

**DOI:** 10.1016/j.psj.2024.103710

**Published:** 2024-04-02

**Authors:** Raul Hector Marin, Jorge Martin Caliva, Jackelyn Melissa Kembro

**Affiliations:** ⁎ Universidad Nacional de Córdoba (UNC), Facultad de Ciencias Exactas, Físicas y Naturales, Instituto de Ciencia y Tecnología de los Alimentos (ICTA), Córdoba, Argentina; † Consejo Nacional de Investigaciones Científicas y Técnicas (CONICET), Instituto de Investigaciones Biológicas y Tecnológicas (IIByT, CONICET-UNC), Córdoba, Argentina

**Keywords:** poultry welfare, behavioral dynamic, robot sensor, environmental enrichment

## Abstract

Welfare problems in broiler chickens are associated with accelerated growth in high density and barren environments. Encouraging broiler movement yields benefits by increasing locomotion, foraging, and environmental exploration. Robot sensors with autonomous navigation capabilities developed to collect husbandry information could collaterally induce movement of birds while traversing the chicken houses. This study examines the short-time dynamic of changes in broiler spatial distribution within the robot's zone of influence throughout the growing cycle. Two batches of mixed-sex Cobb-500 were raised in a commercial broiler farm until 42 d of age, in 2 houses divided into 4 equally sized sectors. In half of the sectors an AviSense robot sustained 2-h per day of autonomous navigation. The minute prior and the 4 min following the robot entering the zone of influence were video recorded weekly. Control sectors without a robot were analyzed equivalently. Number of individuals within the zone of influence of the robot were obtained at 1-s intervals and relative density (%) was estimated. Physical interactions between broilers and the robot, as well as interactions with the environment were also recorded. The entrance of the robot triggers within seconds a strong depopulation of the zone with birds walking to neighboring areas (*P* < 0.03, in all ages). The decreases in relative density induced by the robot appears more pronounced, and repopulation of the zone was slower, in younger than in older birds (*P* < 0.05). Broilers´ showed physical interactions towards the robot and were also touched and/or slightly pushed by the robots (19 and 84% of videos recorded, respectively). They were also found scratching and/or pecking the ground after the robot passed (64% of videos). Findings strongly suggest that robots, beyond their specific capabilities as environmental sensors, were effective in promoting increased movement in broilers along the growing cycle and could also favor additional exploratory behaviors. Thus, these robots could be considered as environmental enrichment elements that contribute to welfare improvements during intensive rearing.

## INTRODUCTION

Welfare problems in broiler chickens are primarily associated with their accelerated growth, high density during rearing (more than 32 kg/m^2^ at the time of slaughter), and the lack of environmental enrichment (Bessei, 2006; Riber et al., 2018). In just 42 d, there is an increase from approximately 50 grams at hatch to over 3 kg (Knowles et al., 2008). This rapid weight gain coupled with limited space to move and an environment lacking stimuli beyond basic activities such as eating, drinking, and resting, may leads to substantial health consequences and disorders (Knowles et al., 2008). For example, there is evidence of the development of long bones and an incidence of locomotion problems ranging from 3 to 27% at the time of slaughter (Knowles et al., 2008), undoubtedly affecting the welfare of the birds (Santos et al., 2022). It is well-established that a key factor in improving the health of the skeletal (and associated muscular) system in broilers is to promote an increase in the levels of physical/locomotor activity (Hong et al., 2024; Shipov et al., 2010).

Over the last decade, the application of precision livestock farming to poultry farming systems has introduced novel technologies capable of offering real-time and continuous overview of the status of birds. This can enable rapid interventions beneficial for the flocks (Rowe et al., 2019; Sassi et al., 2016). Among the variables proposed for monitoring at the environmental level are temperature, humidity, and ammonia levels. This monitoring is not limited to central points within the sheds, which represent an average value. Instead, multiple points are included, thereby better representing the real variability that can exist within the rearing sheds. At the animal level, the measurement of variables associated with weight, behavior, space utilization, lameness, and unplanned deaths have also been proposed (Rowe et al., 2019; Ren et al., 2020; Ojo et al., 2022). As a strategy, technologies are now combining sensors with machine learning algorithms and/or computer vision within the framework of the Internet of Things (**IoT**) (Ojo et al., 2022). However, compared to general agricultural activities, new technologies, including robotics applications, are still relatively scarce in poultry farming (Ren et al., 2020). Only in recent years have some companies offering this technology entered the market. Robots with multiple sensors include the ChickenBoy type (Octopus Robots, 2019) that uses significant shed roof structures to move along the house to register air quality parameters, estimates the ground-level temperature and humidity, and identifies sick and dead animals by temperature (Hartung et al., 2019). Interestingly, robots equipped with autonomous navigation capabilities, designed for ground-level movement, have also been developed (Usher et al., 2015). This technology shows great potential, particularly in broiler production, as these birds are also routinely raised at floor level. For example, the Octopus robot has been proposed for the disinfection of sheds without human intervention through the automatic mixing and ventilation of the birds' bedding (Octopus Robots SA, 2019). Also, in laying hens, the Spoutnic robot aims to address floor egg-related issues by "training" birds to lay eggs in nests (TIBOT Technologies, 2019). However, scientific publications validating these commercial proposals are scarce, and therefore, their effectiveness has not yet been fully evaluated (Usher et al., 2015; Parajuli et al., 2020a; Parajuli et al., 2020b). Although the movement of the robot could be initially considered as a disturbance that might induce stress, an increase in exposure to robots from bi-weekly to daily has been shown to decrease avoidance distance (Parajuli et al., 2020a). Thus, considering habituation processes (Jones, 1996; Nazar and Marin, 2011) if broilers are exposed to the robot beginning at early ages, the flock could potentially quickly acclimate to their activity. Interestingly, it has been shown in broilers that a robot activity does not induce more stress than non-invasive maintenance human activities (Parajuli et al., 2020a).

A new AviSense robot has been developed and is continuously undergoing refinement. It is equipped to monitor shed temperature and humidity by traversing the farm through defined transects to detect environmentally problematic points. During that task, it also takes birds´ pictures aiming to estimate broilers´ growth. Interestingly, while records are being taken, the activity of the robot appears to encourage birds to move from their quasi-static positions. As mentioned above (Shipov et al., 2010; Hong et al., 2024) encouraging broiler movement should yield several benefits, including heightened locomotor activity, foraging, and environmental exploration. Furthermore, favoring birds’ displacement and activity is also associated to an improved bedding quality and welfare associated benefits (Nicol et al., 2017; Widowski and Rentsch, 2022). Obviously, these anticipated behavioral advantages need a careful and thorough evaluation to validate predictions and fine-tune procedures. Fine-tuning may involve regulating the robot's travel speed, appearance, and/or incorporating conspicuous objects that elicit socially favorable behavior as the robot navigates the chicken facility. This initial study aims to examine the short-time dynamic of changes in broiler spatial distribution within the operational range of a robot throughout the growing cycle. We hypothesize that birds will engage with robots and that the robot´s activity will induce spatial movements among the birds within the house areas. To test this hypothesis, video recordings of sectors of the house where birds were exposed to a robot were compared to separate control sectors where no robot was present. A computer vision model was implemented to automatically count the number of birds within the robot´s operational range at high-resolution intervals throughout the study period.

## MATERIALS AND METHODS

The study complies with applicable Argentinean laws, with the local Argentinean Association for Science and Technology Laboratory Animals − (**AACyTAL** Bulletins number 15 and 16, 2001) and was approved by the Institutional Committee for the Use of Laboratory Animals (**Acta 023**). Birds were also reared under commercial following Cobb guidelines and the Argentinian SENASA (Servicio Nacional de Sanidad y Calidad Agroalimentaria) regulations.

### Animals and Husbandry

Mixed-sex Cobb-500 chicks were obtained from a commercial hatchery (INDACOR S.A.) and reared to 42 d (age at slaughter) at a commercial broiler farm operated by INDACOR. The study was carried out in 2 identical chicken rearing houses, each measuring 200 m x 12 m, with an equal distribution of sexes and each divided into 4 equally sized sectors (50 m x 12 m) using wire mesh fences to restrict movement of birds between sectors. The houses were equipped with blackout side curtains, fan-ventilation and an evaporative cooling system. Water and feed (starter and grower rations depending on age) were supplied ad libitum throughout the experiment using feeders and drinkers positioned along feeding and drinking lines. The maximum stocking density recorded was 32 kg/m^2^.

### Robot Description, Video Recording and Study Design

**Figure 1:**
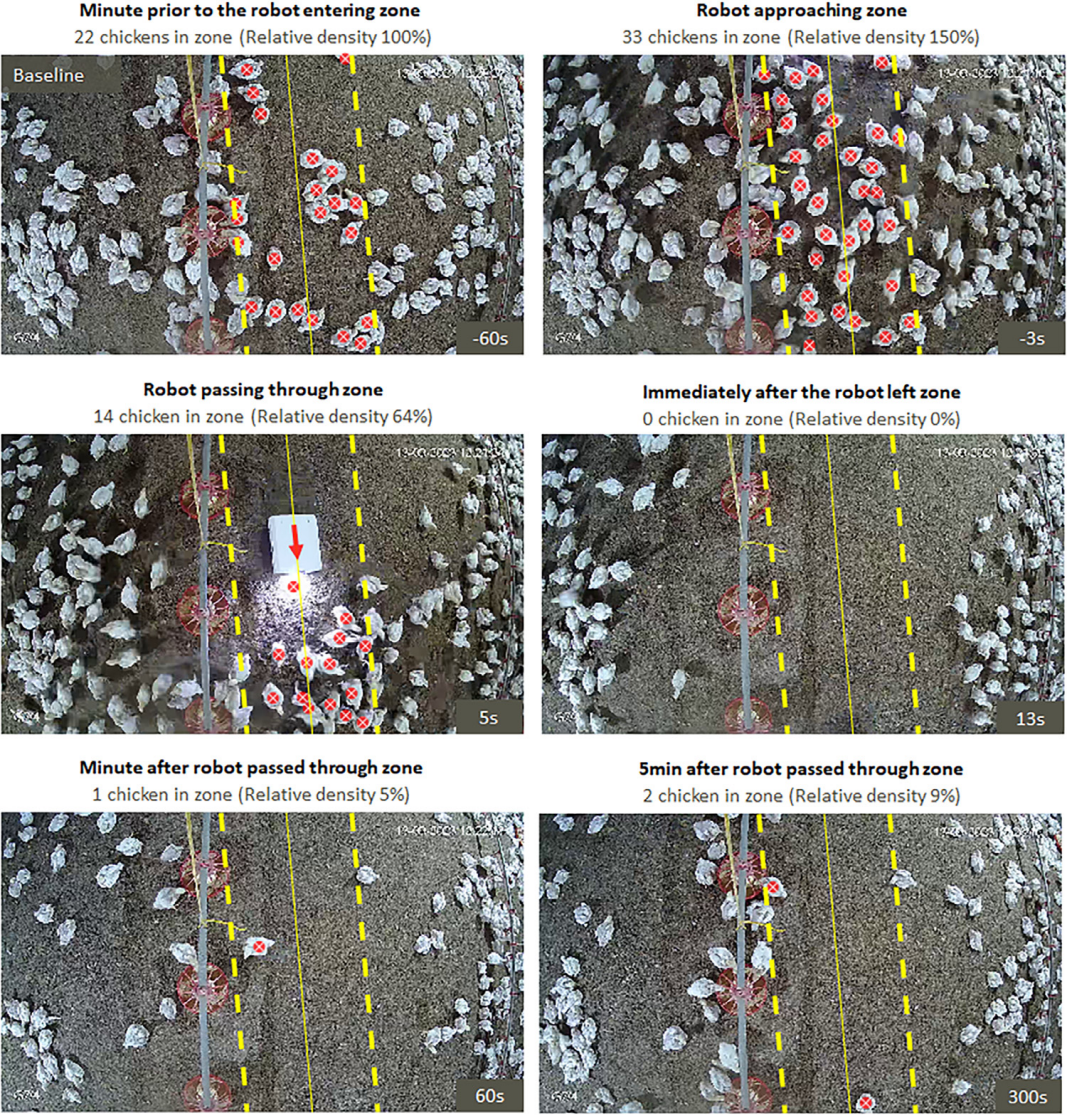
The dynamic impact of the robot on chicken density within its immediate zone of influence. The immediate zone of influence is centered on the trajectory of the robot, represented by the thin yellow line. Bird detection was automated through a customized computer vision model. Chickens, identified by their center of mass marked with a white “X” within red circles, were counted if their center of mass was within the designated zone. Relative density was calculated as the percentage of birds within the zone compared to baseline values obtained before the robot entered the zone (baseline, first image).

AviSense robot vehicles (Figure 1) from Appelie Robotics (APPELIE S.A.S., Córdoba, Argentina) were chosen for this study. The robots measured 38 × 31 × 16.2 cm (L x W x H) and weighed 6.2 kg with a ground clearance between 4 to 8 cm. A front white light consisting of four 10 mm LEDs (9,000-10,000K; 3W) provided a ray of light of 260 to 280 lumens at an angle of 120 to 140°. Robots are provided with a navigation system that allows autonomous but programmable routes through the chicken facility at a maximum speed of about 0.2 m/s. Two sectors per house received a robot. These robots were operated during daylight hours from d 7 until the end of the study (6 wk of age) and were programmed for 2 h per day of effective movement. The programmed movement was continuously along predetermined transects positioned between the water and feeding lines (Figure 1). This approach allows completion of the transect route through the sector within a 1-h period. The robot traveled the total distance from one side to the other side of the house sector assigned to robot treatment (100% of the length; i.e., 50 m). Birds in the remaining 2 sectors per house were never exposed to robots and served as controls. The experiment was repeated across two batches, with each batch involving the rotation of the robot-traversed section within each house to balance potential environmental clues.

Video recordings were obtained weekly within all sectors (with and without robot activity) along the growing cycle, in the morning between 10 and 13 am. At each of the weekly time points, 5-min videos were recorded at 25 frames per second and saved in MP4 format with a resolution of 1,280 × 720 pixels. Ideally, recordings included at least the minute prior to the robot entering the camera's field of vision and the following 4 min after the robot left the field. Due to camera or recording failures, some videos were lost or too short to be considered for analysis, thus for dynamics a total of 75 videos were analyzed, with at least 5 replicates within each condition.

Video recordings were also inspected to register potential physical interactions between the broilers and the robot. Whether or not a given behavior (see definitions below) was observed by any bird within the camera's field of vision was recorded, as well as the number of birds involved in the interaction. Interactions initiated by the birds towards the robot were defined as follows:

­ Close approaching robot: during the video recording at least one bird followed the robot within a 5 cm zone.
­ Pecking at the robot: during the video recording at least one bird intentionally physically touched the robot with its beak.
­ Jumping onto the robot: during the video recording at least one bird stood with both feet on top of the robot.

Interactions initiated by robot towards the birds were defined as:

­ Birds touched by the robot: when any side of the robot makes contact with a bird, whether the bird is still or in motion, it does not induce involuntary movement in that bird.
­ Birds pushed by the robot: when the front side of the robot makes contact with a bird during movement, and induces an involuntary movement in that bird.

Videos showing birds interacting with the environment after the robot passes were also registered as follows:

­ Scratching the ground: at least one bird used its feet to move the litter within 4 minutes after the robot had passed over it.
­ Pecking the ground: at least one bird pecked at the litter within 4 minutes after the robot had passed over it.

Results were presented as a percentage of videos in which interactions were found.

In addition, changes in the number of birds within the zone of influence of the robot (or an equivalent zone in control sectors) were evaluated in each video recording at a 1 s sampling intervals. For this, video-recordings were converted into images at 1 s intervals for behavioral analysis and variable estimation. As detailed below, these images were analyzed automatically using a computer vision model based on Li et al (2023). Specifically, the oriented object detection model, You Only Look Once V8 (**YOLOv8**) (Jocher et al., 2023) was used (see Section Analysis of videos using computer vision model). The YOLO is a popular object detection model with stable and accurate detection performance that has been previously used for detecting poultry in images (Doornweerd et al., 2023; Guo et al., 2023; Li et al., 2023). The computer vision model required prior training and validation for which a separate set of images were obtained from videos (see next section). Code is freely available on Figshare (Brito, 2024).

### Training and Validation of Computer Vision Model for Chicken Detection in Images

One hundred and ten images were randomly selected for the training and validation set, representative of each week of age and sector, and included images with and without a robot present. A total of 7989 chickens were labeled from 63 images without robot and 4,325 from 47 images with the robot. Labeling for object detection (i.e., chickens, robot, feeders) was performed in RoboFlow, using horizontal rectangular bounding box. The dataset was split into 60% for training, 27% for validation, and 13% for testing. The model was trained and validated on Roboflow using the web interface. Preprocessing steps only included auto-orient and resizing (stretch to 800 × 600). No augmentations were applied.

### Performance Evaluation

Precision, recall, F1 score, and the average precision metric (**mAP**) estimated across all classes in model were used to determine the final performance of oriented object detection and calculated.

Precision(%)=(100×Truepositives)/(Truepositives+Falsepositives)

Recall(%)=(100×Truepositives)/(Truepositives+Falsenegatives)

F1score=2×((Precision×Recall)/(Precision+Recall))

$$Average\;precision\;metric\;=\frac{1}{N}\;\sum_{i=1}^{N}AP_{i}$$

where True positives are number of cases in which both algorithms and labels show bird presence; False positives is number of cases in which algorithms wrongly predicted bird presence; and False negatives is number of cases in which algorithms wrongly predicted bird absence. In the average precision metric formula, AP is the average precision for each class (i.e., chickens, robot, feeders) and then averaged over the number of classes (i.e., N = 3). For estimations, an intersection over union (**IoU**), indicating the overlap of the predicted bounding box coordinates to the labeled box (ground truth) was 0.3, and the confidence level 50. The developed computer vision model was saved as a .pt file for further behavior analysis. A precision of 90.4%, a recall of 84.9%, F1 score of 87.6 and average precision metric value of 92.2% were determined.

### Analysis of Videos Using Computer Vision Model

The computer vision model was deployed in Phython's Integrated Development and Learning Environment (Phython 3.11.6) in a local machine with the processor Intel Core i7-8700 CPU @ 3.2 GHz, RAM 16 GB. Each image extracted from video recordings underwent processing through the trained and validated computer vision model. The model generated detailed information in the form of rectangular bounding boxes encompassing each object (i.e., feeder, robot, and chicken), including the specific parameters X_min_, Y_min_, box width, and box length. Furthermore, the coordinates for the center of each bounding box were computed on both the X- and Y-axis, representing the centroid coordinates for every object within the image.

For each sector of the rearing houses at each age point, the robot's centroid coordinates were utilized to establish the trajectory of the robot (continuous yellow lines in Figure 1). Subsequently, the zone of influence of the robot was arbitrarily defined as 1.5 times the width of the robot on either side of the trajectory (thick dotted yellow lines in Figure 1). Chicken whose centroid coordinates fell within the zone of influence were counted (white “X” within red circles in Figure 1) at each 1 s time point. Ideally, the analysis covered 1 min before and 4 min after the robot passed through the camera´s field at 1 s intervals. For the control sections, without a robot, similar zones and time frames were employed.

To facilitate comparison, given the variations in bird size and density throughout the experiment, relative density was estimated as the percentage of birds at each time point in relation to baseline values. Baseline values were computed as the mean number of birds in the zone of interest between 15 and 60 s before robot detection.

For statistical analysis (see next section for details) relative density data was estimated at three time points: the moment prior, the minute after and 2 min after the robot enters the camara's field of vision. To account for variability in the assessment of a potential increase in the number of birds within the camera's field of vision induced by the entry of the robot, the variable “relative density at the moment prior” was estimated as the maximum value of relative density within the 15 s period before the robot entered the camera's field of vision. For the following two time points, the relative number of birds estimated at one or two minutes after the robot enters the camara's field of vision were recorded.

### Statistical Analysis

Physical interactions between the broilers and the robot, as well as interactions of birds with the environment, were analyzed using proportion tests that evaluated whether the proportions of videos showing these interactions differed from zero occurrence.

Relative density data at 3 time points, the moment prior, the minute after and two minutes after the robot enters the camara's field of vision, were evaluated using Generalized Lineal Mixed models (**GLMM**) taking into account the distribution of each variable. For the moment prior, the best fit included the use of a gamma distribution, while for the other two time points a normal distribution with data transformed into ranks were used (Shirley, 1987). The model included treatment (with or without robot) as fixed effect and batches as a random effect. Age was not included in the model as a fixed factor because we could not guarantee that the same birds were sampled at all ages. A P-value of < 0.05 was considered to represent significant differences.

To assess potential differences along the growing cycle (age) on the 3 time points (prior, and 1 and 2 min after the passage of the robot), relative density data from the first and last week of growing were compared. A GLMM was employed, incorporating treatment (with or without robot) and age (1st or 6th wk) as fixed effects, along with the interaction between these factors. Additionally, batches were considered as a random effect. Considering the 6-wk interval between these data collection points, they were considered in the model as if they were independent. Linear regressions between the relative density data and time after the passage of the robot were performed to estimate full repopulation time points. The coefficient of determination (R^2^) was subsequently calculated.

## RESULTS

Figure 1 and Supplementary Video 1 illustrate an instance of chicken displacement induced by the robot. In this example, just a few seconds before the robot enters the camera´s field of vision, there is a noticeable increase in the number of chickens, attributed to animal movements induced by the approaching robot (Figure 1, top right panel). Following the robot´s passage, the zone of influence becomes nearly depopulated of birds and even 5 min afterwards, only few birds are observed within the zone (Figure 1, Supplementary Video 1.

**Figure 2:**
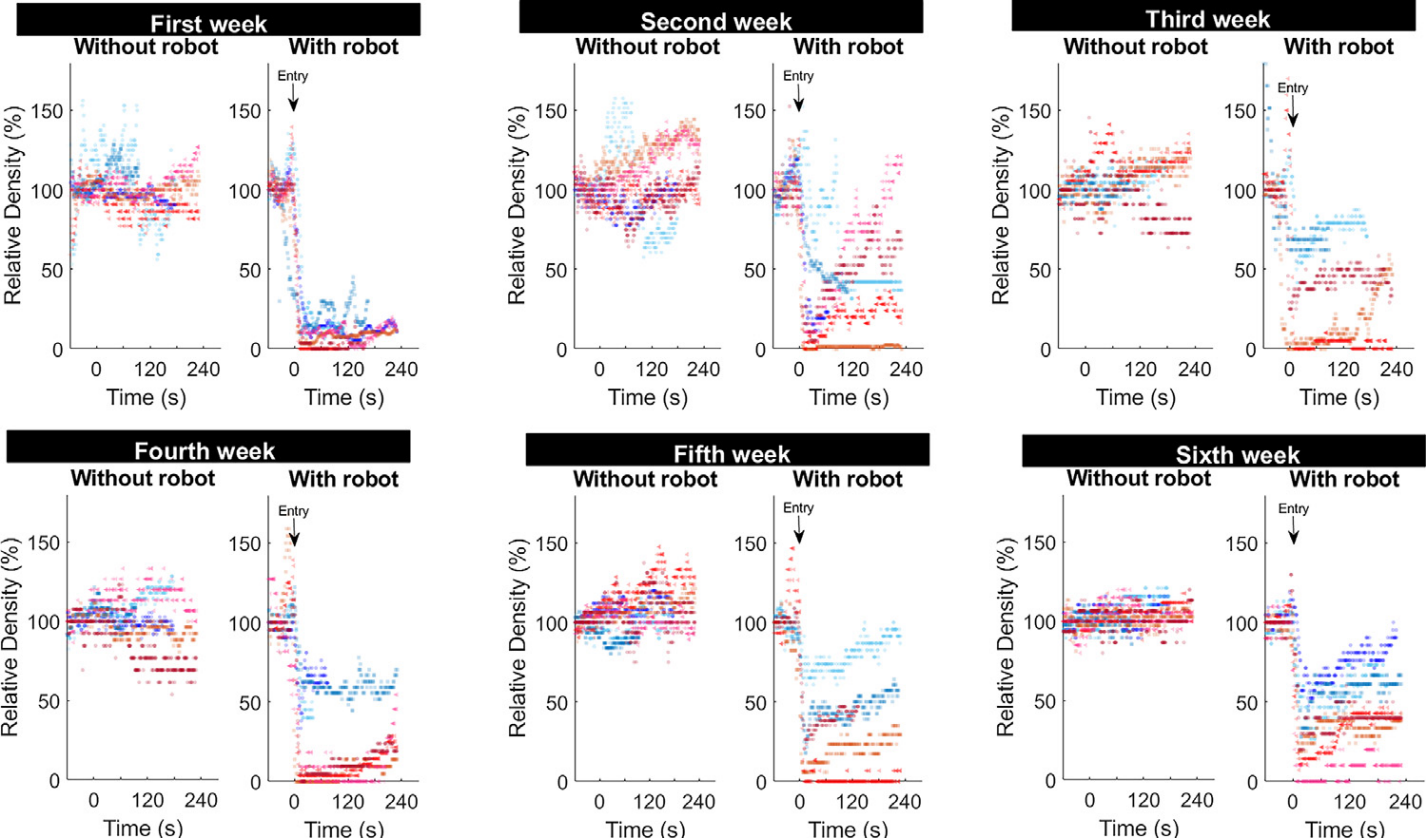
Variability in the dynamic impact of the robot on chicken density over time. Each data point represents the relative density of birds within the robot´s zone of influence, ranging from 1 min prior (-60 s) to four minutes (240 s) after the robot´s s entry into the zone, sampled at 1 s intervals. While temporal variability is evident in all groups (with and without robots), a drastic reduction in relative density is only observed immediately after the robot enters the zone (indicated by the arrow), consistently across all cases. Each color represents a replica of the study with blues and reds representing batch 1 and batch 2, respectively.

Figure 2 depicts the short-time dynamic impact of the robot on chicken density over the 6 wk growing cycle in all sectors studied. An increase in relative bird density before the robot enters the camera's field of vision (Figure 2 time point indicated with an arrow) was not consistently observed, as evidenced in a few replicates. However, it was found to be statistically different from controls (*P* = 0.006) at the 2nd wk of age (Table 1). Noteworthy, a remarkable reduction in the relative density was found consistently (*P* < 0.03, in all cases) both, 1 and 2 min after the robot traversed the zone of influence at all ages evaluated (Figure 2; Table 2). Furthermore, across nearly all groups and ages, although an initial repopulation of the robots´ zone of influence was evidenced, the relative density remains notably lower compared to controls for up to 4 min following the passage of the robot (Figure 2).

**Table 1:** Relative density of birds (%) within groups tested with and without robot, at the moment prior to the robot entering the camara's field of vision.

Wk	Moment prior		
	Without robot	With robot	*P*-value
1st	108.9 ± 4.4^a^	113.2 ± 10.6^a^	0.74
2nd	104.3 ± 4.6	122.1 ± 3.6	0.01
3rd	107.7 ± 4.3	103.9 ± 25.2	0.91
4th	108.9 ± 2.8	121.2 ± 9.6	0.26
5th	105.9 ± 2.1	114.0 ± 8.3	0.17
6th	105.7 ± 2.1^a^	112.9 ± 3.9^a^	0.10

Mean ± SEM and estimated *P*-values (*P*) are shown. Sample size (n) varied between 5 and 7. wk: week of age. The moment prior was considered as the maximum value of relative density within the 15 s period before the robot entered the camera's field of vision.

a Independent statistical analyses performed including only the 1st and 6th wk of age did not show effects of robot treatment, age or the interaction between factors.

**Table 2:** Relative density of birds (%) within groups tested with and without robot one and two minutes after the robot enters the camara's field of vision.

Wk	Minute after			Two min after		
	without	With	*P*-value	Without	With robot	*P*-value
1st	104.8^a^ ± 7.8	10.8 ± 3.7^b^	0.002	93.2 ± 4.8^a^	9.1 ± 4.5^b^	0.004
2nd	105.0 ± 11.0	34.7 ± 11.0	<0.001	99.4 ± 10.4	35.8 ± 12.3	0.003
3rd	109.7 ± 5.6	41.3 ± 16.3	0.003	115.1 ± 2.5	34.8 ± 17.4	0.030
4th	103.6 ± 5.3	24.0 ± 14.0	<0.001	112.0 ± 7.0	14.8 ± 9.4	0.010
5th	101.9 ± 4.3	33.9 ± 12.4	<0.001	109.2 ± 4.7	38.6 ± 13.6	0.004
6th	103.5^a^ ± 2.1	38.8 ± 9.0^c^	<0.001	104.6 ± 2.3^a^	47.1 ± 10.1^c^	<0.001

Mean ± SEM and estimated *P*-values are shown. Sample size (n) varied between 5 and 7. wk: week of age. Independent statistical analyses performed including only the 1st and 6th wk of age showed interactive effects with robot treatment, both one and two minutes after the robot enters the camara's field of vision.

a-c Within each time point, conditions that do not share the same letter differ with *P* < 0.05.

Supplementary Video 2 shows an example of physical interactions initiated by birds towards the robot. A significant proportion of videos showed physical interactions between the broilers and the robot, as well as interactions of birds with the environment (*P* ≤ 0.05, in all cases). Specifically, birds´ showing interactions towards the robot, close approaching and/or pecking at it, were observed in 19% of the videos recorded during the first 3 wk of age. No jumping onto the robot was observed during the whole study. Interactions initiated by the robots (robot touching or slightly pushing birds) were found in 84% of the videos recorded and across all ages. On average, 4.0 ± 0.6 birds (ranging from 1.6 ± 0.7 at the 1st wk to 5.0 ± 1.6 at the 6th wk) were touched by the robot as it passed through the zone of interest. Birds were also found interacting with the environment (i.e., scratching and/or pecking the ground) after the robot passed in 64 % of the videos.

Comparisons between the relative density at 1st and 6th wk of age both one and two minutes after the robot traversed the area showed age, robot and interactive effects at *P* < 0.001 in all cases. Post-hoc analyses (^a-c^letters in Table 2) showed that relative density remained close to 100% in all control groups without robots independently of age. However, birds exposed to the robots showed higher relative density (*P* < 0.05), both one and two minutes after the robot passed in the 6th wk (39 ± 9% and 47 ± 10, respectively), compared to the 1st wk of age (11 ± 4% and 9 ± 5, respectively). No age-related robot influences were detected before its entry (Table 1).

**Figure 3:**
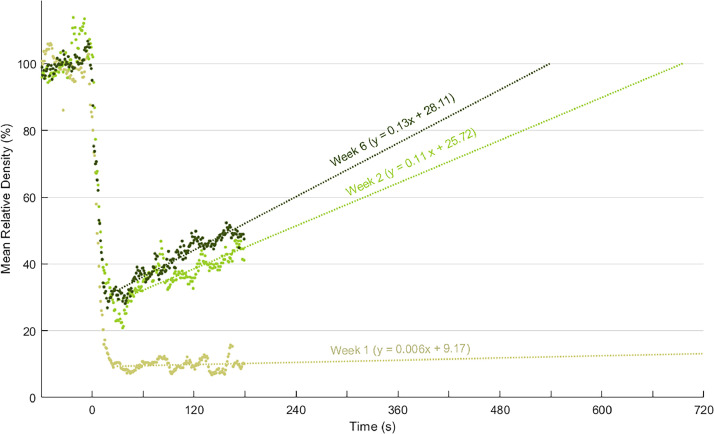
Dynamic of repopulation across different ages. Mean values of relative density for each age were estimated from the values shown in Figure 2. A linear regression model was fitted to mean values of relative density for each age group. To avoid cluttering only 1st, 2nd and 6th wk of age were plotted.

Mean relative density data was used for theoretical estimations of the time required for birds to repopulate the zone of influence using a linear regression model. Mean group values over time, as well as the linear regression model estimated for the 1st, 2nd and 6th wk of age, are shown in Figure 3. Interestingly, repopulation of the zone of influence was remarkably slow during the 1st wk of age, with the estimated slope approaching zero, hence indicating a lack of repopulation during the evaluated time frame. However, at 2nd wk and at the end of the growing cycle (6th wk), significant positive slopes were observed (*P* < 0.001). A slightly steeper slope was observed on the 6th wk (0.13; R^2^ = 0.87) compared to the 2nd wk (0.11; R^2^ = 0.65), as shown by the fitted lines depicted in Figure 3. These slopes denote a progression wherein the robots´ zone of influence would be fully repopulated faster for older birds than for younger birds (Figure 3).

## DISCUSSION

This study focusses on examining short-time dynamic of changes in broiler spatial distribution within a robot's zone of influence throughout the growing cycle. It is evident that the proximity of the robot to the birds triggers a strong depopulation of the zone within just a few seconds. Birds are therefore induced to move to neighboring areas. Throughout the growing cycle, the decreases in relative density induced by the robot appears more pronounced in younger birds than in older ones. Conversely, the repopulation after robot passes shows the opposite pattern, with older birds repopulating the area faster than younger birds.

The effects of the robot can be observed, in some opportunities, even before the robot enters the camera's field of vision, as a brief increase in relative bird density occurred just a few seconds prior to its entry (Figure 1, Figure 2, Supplementary Video 1). However, on average, this increase was only found to be different from controls at the 2nd wk of age. This eventual robot´s “entrance” effect, as well as the general depopulation of the zone of influence of the robot, can initially be explained by two mutually inclusive alternatives. Firstly, it could be due to the effect of the robot's approach on the birds (i.e., push or touch effect), where animals are compelled by physical contact to move from their positions. The second alternative could be due to a fear response induced by novelty, as has been shown with other unfamiliar inanimate stimuli (Jones, 1996). In this scenario, birds not habituated to the robot presence would distance themselves from the robot, avoiding contact in advance due to fear (i.e., fear effect), as with avoidance to humans (Usher et al., 2015; Parajuli et al., 2020a). It is foreseeable that at the beginning of the growing period, a greater displacement induced by fear effect would be observed. As the birds grow, fear would decrease as they become accustomed to the robot's passage (ceasing to be unfamiliar). In line with this contention, not only the relative density was decreased less but also the repopulation occurred faster on the 6th wk compared to the 1st wk of age. Also consistently, the study conducted by Parajuli et al. (2020a) in broilers, showed that even a brief daily stimulus with a robot, results in a gradual decrease over time in the avoidance distance to the robot. Furthermore, their study also showed shorter avoidance distances when the robot was operational on a daily rather than on a biweekly basis, suggesting that a higher frequency of exposure to the robot favors an habituation process (Parajuli et al., 2020a). In our study, the robot was programmed for 2 h per day of effective movement; therefore, an even faster habituation process was expected. Interestingly, 84% of our video recordings showed instances where birds were touched or even slightly pushed by the robot, indicating null avoidance distances. Additionally, during the first 3 wk of age, when broilers are more active, few birds were also observed seeking physical interactions with the robot by approaching and even pecking at it. Thus, behavioral findings further support a habituation process where novelty is reduced via regular exposure to the same objects (Jones and Waddington, 1992; Jones, 1996) especially since it was provided at early ages (Skånberg et al., 2023). In fact, from the 2nd wk onward, there were no significant increases in bird density before the robot´s entrance, suggesting that the robot's movement induces a slightly “pushing” or gentle “get out of the way” effect rather than a response driven by fear-induced avoidance. Taken together, these findings suggest that birds are capable of quickly habituating to robots, and fear-induced reactions do not appear to underlie the observed behaviors after habituation. The reduction of any initial fear response is highly relevant given that although fear responses have adaptive properties for survival, in intensively housed poultry they should be avoided to minimize potential harm effects on welfare and performance (Jones, 1996).

Broilers birds are highly social animals, and it is well known that they tend to remain in close proximity to conspecifics, exhibiting a cluster like behavior (Febrer et al., 2006; Estevez, 2007). Hence, the spatial distribution displayed in groups can show a non-random spread over the available space if the environmental conditions allow such spreading. The data on the observed ambulation dynamics of the birds after the passage of the robot, along with theoretical calculations based on linear regression, indicate a progression wherein the zone of influence of the robots would be fully repopulated faster for older birds than for younger birds. This is particularly noticeable during the 1st wk, where repopulation is negligible during the period evaluated. This age effect could be in part due to low density of broilers (kg/m^2^) at the beginning of the growing period, leaving large amounts of floor area unoccupied. At this early age, animals could simply move from their original positions to other areas of the poultry house. Subsequently, they may remain active and grouped in those areas, likely until new stimuli prompt further movements, such as a new passage of the robot. As mentioned above, some birds at early ages were found actively following and engaging in physical interactions (pecking) with the robot (Supplementary Video 2). Thus, early exposure to the robot, since newly hatched chicks are initially brooded, could have a noteworthy collateral effect if imprinting processes are developed. Imprinting is a behavioral phenomenon that fosters early and strong birds´ attachments to the first moving objects encountered, establishing a bond associated with enhanced sense of security, increased ambulatory behaviors, reduced stress responses, and consequently, an improved welfare of the imprinted birds (Salvatierra et al., 1994; Tzschentke and Plagemann, 2006). Nevertheless, specific studies should be carried out to test the potential utilization of sensor-equipped robots as imprinting stimuli.

It is important to note that age is also associated with substantial weight gain, potentially causing the birds to be less active and discouraged to move in the presence of the robot (Parajuli et al., 2020a). Moreover, the open floor space in the broiler house became filled as broilers got older and larger, which in turn restricts the birds’ field-of-view and movement (Parajuli et al., 2020a). Herein, on the 6th wk of age, the robot touched on average 5.0 ± 1.6 birds while passing through the zone of interest. Given the elevated density at which the birds are raised, the impact initiated by the robot can be likened to a domino or herding-like phenomenon. In this scenario, the robot interacts with a select few birds positioned in its trajectory, prompting these individuals to vacate their locations. Subsequently, this sequential displacement can also influence other neighboring flock mates. The remarkable reduction in the relative density found consistently after the robot traversed the zone of influence at all ages evaluated suggest that the robot is capable of inducing a displacement of the birds and, consequently, promotes their locomotor/ambulatory activity. This increased activity could be of importance since it is well-established that a key factor in improving the health of the skeletal (and associated muscular) system in broilers is to promote an increase in the levels of physical/locomotor activity (Shipov et al., 2010; Hong et al., 2024).

Repopulation of the zone of interest is slow and could take more than 8 min (Figure 3) even on the 6th wk. The fact that the birds' bedding remains clear for at least a few minutes can also be considered beneficial because it could contribute, even if minimally, to improving the ground condition by allowing ventilation. Furthermore, when the birds repopulate that space, a promotion of additional beneficial behaviors, such as ground scratching and foraging explorations would be expected. Indeed, our study also found, in 64.8% of the videos, birds interacting with the environment by scratching and/or pecking at the bedding that had been depopulated after the passage of the robot. These behaviors, in turn, may contribute to improvements in bedding quality, as they are known to positively influence foot and breast health, moisture control, thermoregulation, ammonia reduction, and overall management practices, including waste removal and cleaning (Nicol et al., 2017; Widowski and Rentsch, 2022). Nevertheless, further specific experimentation needs to be done to ensure that the proposed beneficial consequences on broiler welfare (muscle/skeletal and bedding related), are indeed confirmed.

Poultry broiler houses are often considered as relatively barren environments that certainly have detrimental effects on the expression of behavioral needs (Widowski and Rentsch, 2022). In this context, our findings suggest that robots could serve as a valuable tool for environmental enrichment. The presence of robots not only encouraged increased movement in birds but could also lead to an increase in additional behaviors, such as ground scratching and foraging explorations.

It would also be intriguing to explore in the future whether changes in the features (shape, colors, etc.) of the robots throughout the rearing process contribute to sustaining increased motor activity over time. While it might be posited that novel elements could potentially induce prolonged fear responses, as mentioned above, fear-induced reactions do not appear to underlie the behaviors induced by the presence of the robot. Indeed, studies have already demonstrated that positive interactions with new elements can also lead to favorable cognitive adjustments, thereby reducing stress reactions to new stimuli (Riber et al., 2018; Tahamtani et al., 2018).

In conclusion, the present findings show that the passage of the robot within the poultry house induces a dynamic shift in the spatial distribution of broilers, leading to immediate displacements of birds from their current locations to neighboring areas. Subsequently, the birds start repopulating the spaces they had temporarily vacated. Additionally, it has been noted that the robot not only promotes broilers´ locomotor activity but may also elicit exploratory behaviors on the bedding upon their return to previously vacant spaces. The rise in locomotor activity induced by the robot suggests its potential to enhance bird welfare by promoting skeletal health and associated muscular systems, along with an increase in associated exploratory behaviors. The observation that the birds' bedding remains clear for at least a few minutes can also be considered advantageous as it may contribute to bedding ventilation. In summary, these findings strongly suggest that robots, beyond their specific capabilities as environmental sensors, should be regarded as environmental enrichment elements, fostering welfare improvements during intensive rearing.

## Appendix Supplementary materials

**Supplementary Video 1.** Example of the dynamic impact of the robot on chicken density within its immediate zone of influence (compare with Fig. 1). Image, video 1

**Supplementary Video 2.** Example of interactions between broilers and the robot. Image, video 2

## Footnotes

Supplementary material associated with this article can be found in the online version at doi:10.1016/j.psj.2024.103710.

## References

[bib0001] Bessei W. (2006). Welfare of broilers: a review. World's Poult. Sci. J..

[bib0002] Brito, D. 2024. Code for chicken and robot detection using Yolo v8, figshare. Accessed Apr. 2024. 10.6084/m9.figshare.25563072.

[bib0003] Doornweerd J.E., Kootstra G., Veerkamp R.F., de Klerk B., Fodor I., van der Sluis M., Bouwman A.C., Ellen E.D. (2023). Passive radio frequency identification and video tracking for the determination of location and movement of broilers. Poult. Sci..

[bib0004] Estevez I. (2007). Density allowances for broilers: where to set the limits?. Poult. Sci..

[bib0007] Febrer K., Jones T.A., Donnelly C.A., Dawkins M.S. (2006). Forced to crowd or choosing to cluster? Spatial distribution indicates social attraction in broiler chickens. Anim. Behav..

[bib0008] Guo Y., Aggrey S.E., Yang X., Oladeinde A., Qiao Y., Chai L. (2023). Detecting broiler chickens on litter floor with the YOLOv5-CBAM deep learning model. Artif. Intell. Agric..

[bib0009] Hartung J., Lehr H., Rosés D., Mergeay M., Van Den Bossche J., O'Brien ECPLF B., Hennessy D., Shalloo L. (2019). Pages 272–276 in 9th European Conference on Precision Livestock Farming.

[bib0010] Hong G.A.T., Tobalske B.W., van Staaveren N., Leishman E.M., Widowski T., Powers D.R., Harlander-Matauschek A. (2024). A wing-assisted incline running exercise regime during rearing increases initial flight velocity during descent in adult white- and brown-feathered laying hens. Poult. Sci..

[bib0011] Jocher G., Chaurasia A. Ultralytics YOLOv8. https://github.com/ultralytics/ultralytics.

[bib0012] Jones R.B. (1996). Fear and adaptability in poultry: insights, implications and imperatives. World's Poult. Sci. J..

[bib0013] Jones R.B., Waddington D. (1992). Modification of fear in domestic chicks, Gallus gallus domesticus, via regular handling and early environmental enrichment. Anim. Behav..

[bib0014] Knowles T.G., Kestin S.C., Haslam S.M., Brown S.N., Green L.E., Butterworth A., Pope S.J., Pfeiffer D., Nicol C.J. (2008). Leg disorders in broiler chickens: prevalence, risk factors and prevention. Plos One.

[bib0015] Li G., Li B., Shi Z., Lu G., Chai L., Rasheed K.M., Regmi P., Banakar A. (2023). Interindividual distances and orientations of laying hens under 8 stocking densities measured by integrative deep learning techniques. Poult. Sci..

[bib0016] Nazar F.N., Marin R.H. (2011). Chronic stress and environmental enrichment as opposite factors affecting the immune response in Japanese quail (*Coturnix coturnix japonica*). Stress.

[bib0017] Nicol C.J., Bouwsema J., Caplen G., Davies A.C., Hockenhull J., Lambton S.L., Lines J.A., Mullan S., Weeks C.A. (2017).

[bib0018] Octopus Robots SA 2019. Octopus poultry safe robot (OPS) - © 2019 octopus robots, Accessed Dec. 2022. http://octopusrobots.com/en/home2022.

[bib0019] Ojo R.O., Ajayi A.O., Owolabi H.A., Oyedele L.O., Akanbi L.A. (2022). Internet of things and machine learning techniques in poultry health and welfare management: a systematic literature review. Comput. Electron. Agric..

[bib0020] Parajuli P., Huang Y., Tabler T., Purswell J.L., DuBien J.L., Zhao Y. (2020). Comparative evaluation of poultry-human and poultry-robot avoidance distances. Trans ASABE.

[bib0021] Parajuli P., Zhao Y., Tabler T. (2020). Evaluating avoidance distance and fleeing speed of broilers exposed to aerial systems. Int. J. Agric. Biol. Eng..

[bib0022] Ren G., Lin T., Ying Y., Chowdhary G. (2020). Agricultural robotics research applicable to poultry production: a review. Comput. Electron. Agric..

[bib0024] Riber A.B., van de Weerd H.A., de Jong I.C., Steenfeldt S. (2018). Review of environmental enrichment for broiler chickens. Poult. Sci..

[bib0025] Rowe E., Dawkins M.S., Gebhardt-Henrich S.G. (2019). A systematic review of precision livestock farming in the poultry sector: is technology focussed on improving bird welfare?. Animals (Basel).

[bib0026] Salvatierra N.A., Marín R.H., Arce A., Martijena I.D. (1994). Chick imprinting performance and susceptibility to acute stress associated to flunitrazepam receptor increase. Brain Res..

[bib0027] Santos M.N., Widowski T.M., Kiarie E.G., Guerin M.T., Edwards A.M., Torrey S. (2022). In pursuit of a better broiler: tibial morphology, breaking strength, and ash content in conventional and slower-growing strains of broiler chickens. Poult. Sci..

[bib0028] Sassi N.B., Averós X., Estevez I. (2016). Technology and poultry welfare. Animals.

[bib0029] Shipov A., Sharir A., Zelzer E., Milgram J., Monsonego-Ornan E., Shahar R. (2010). The influence of severe prolonged exercise restriction on the mechanical and structural properties of bone in an avian model. Vet. J..

[bib0030] Shirley E.A. (1987). Application of ranking methods to multiple comparison procedures and factorial experiments. Appl. Stat..

[bib0031] Skånberg L., Newberry R.C., Estevez I., Keeling L.J. (2023). Environmental change or choice during early rearing improves behavioural adaptability in laying hen chicks. Sci. Rep..

[bib0032] Tahamtani F.M., Pedersen I.J., Toinon C., Riber A.B. (2018). Effects of environmental complexity on fearfulness and learning ability in fast growing broiler chickens. Appl. Anim. Behav. Sci..

[bib0034] Tzschentke B., Plagemann A. (2006). Imprinting and critical periods in early development. World’s Poult. Sci. J..

[bib0033] TIBOT Technologies 2019. Spoutnic-©TIBOTTECHNOLOGIES. Accessed Nov. 2022. https://www.tibot.fr/2022.

[bib0035] Usher, C.T., Daley W.D. , Webster A.B. , and Ritz C. 2015. A study on quantitative metrics for evaluating animal behavior in confined environments. ASABE Annual International Meeting, St. Joseph, Michigan, 152190148.

[bib0036] Widowski T.M., Rentsch A.K., Knight A., Phillips C.J.C., Sparks P. (2022). Pages 47–63 in Routledge Handbook of Animal Welfare.

